# Factors Related to Evidence-Based Practice among Community Nurses in Greece: A Cross-Sectional Study

**DOI:** 10.3390/healthcare11233071

**Published:** 2023-11-30

**Authors:** Theodoula Adamakidou, Eleni Ntempeli, Petros Galanis, Alexandra Mantoudi, Christos Kleisiaris, Marianna Mantzorou, Afroditi Zartaloudi, Chrysoula Tsiou, Paraskevi Apostolara

**Affiliations:** 1 Research Laboratory of Home Health Care, Postgraduate Program of “Community and Public Health Nursing”, Nursing Department, University of West Attica, 12243 Athens, Greece; lenadebeli@gmail.com (E.N.); amantoudi@uniwa.gr (A.M.); mantzorou@uniwa.gr (M.M.); vapostolara@uniwa.gr (P.A.); 2 1st Health Center of Peristeri, 12132 Peristeri, Greece; 3 Clinical Epidemiology Laboratory, Faculty of Nursing, National and Kapodistrian University of Athens, 11527 Athens, Greece; pegalan@nurs.uoa.gr; 4 Department of Nursing, School of Health Sciences, Hellenic Mediterranean University, 71410 Heraklion, Greece; kleisiaris@hmu.gr; 5 Nursing Department, University of West Attica, 12243 Athens, Greece; azarta@uniwa.gr (A.Z.); ctsiou@uniwa.gr (C.T.)

**Keywords:** evidence-based practice, community nurse, attitudes, knowledge, practice

## Abstract

Assessing knowledge, attitudes and practices towards evidence-based practice (EBP) is a challenge for healthcare professionals. However, the existing literature focuses on nurses working in acute hospital settings, with nurses working in community and primary healthcare settings receiving comparatively less attention. The purpose of the study was to explore factors that related to attitudes, knowledge and practice of community nurses toward EBP. A cross-sectional study was conducted with a sample of community nurses in Greece. Community nurses around the country were invited to complete an online questionnaire during the period of February to April 2022. To measure nurses’ attitudes, practices and knowledge/skills regarding EBP, the Evidence-Based Practice Questionnaire was used. A total of 164 nurses took part in the study. The mean age of nurses was 41.6 years, with 42.7% having either an MSc or a PhD degree. The overall internal consistency for the EBPQ questionnaire was 0.91. The sample demonstrated a high level of knowledge/skills (mean score of 5.5 on a 7-point scale) and positive attitudes (mean score of 5.5 on a 7-point scale) towards EBP, while the level of EBP practice was moderate (mean score of 4.5 on a 7-point scale). A higher educational level showed a positive relationship with the “practice of EBP” subscale (*p* = 0.005) and the “knowledge/skills about EBP” subscale (*p* = 0.003). Additionally, an increase in the knowledge/skills score was associated with a more positive attitude towards EBP (*p* < 0.001) and better practice of EBP (*p* = 0.003). The identification of educational level as the main factor related to the knowledge/skills and implementation of EBPs in community nurses emphasizes the necessity for educational initiatives in EBP at both undergraduate and postgraduate levels. Informed nurses who are familiar with current guidelines and evidence can effectively train patients about chronic disease management and prevention. Additionally, creating incentives to motivate participation in lifelong learning programs can indeed play a crucial role in enhancing the proficiency of community nurses in evidence-based practice.

## 1. Introduction

The World Health Organization demonstrates that research evidence should form the basis for nursing practice, health and social services, clinical decision making and health policy formulation [1]. Accordingly, the International Council of Nurses places emphasis on the development of research-based professional knowledge [2]. It is precisely this evidence-based practice (EBP) that is concerned with “*the conscious, explicit and judicious use of information based on both theory and research to make decisions about the provision of care to individuals or groups of patients, taking into account their particular individual needs and preference*” [3].

Community healthcare is a highly challenging sector due to resource constraints, insufficient staffing, an aging population and the intricacy of care required [4]. Community nursing focuses on providing healthcare services to individuals, families and communities in various community settings such as the home, schools, health centers and other community-based facilities. Community nurses play a crucial role in promoting health, preventing disease and improving the overall well-being of their communities [5]. Furthermore, the community nursing environment has a degree of autonomy while also requiring collaboration with other healthcare professionals, factors that also need to be taken into consideration when assessing community nurses’ knowledge, attitudes and practices regarding EBP [6]. These factors have positively affected the implementation of EBP compared to employees in other levels of a healthcare system [7].

International organizations and the scientific community are intensely interested in EBP; however, nurses and other healthcare professionals use more practical knowledge based on experience rather than evidence that is the product of research [8]. It has been mentioned that although they are positive about EBP, they are not yet ready to adopt it [9,10]. They often consider experience or advice from colleagues or patients as the most useful source of knowledge or rely on their personal experience from what they have learned in nursing schools, and during the subsequent practice of the profession, rarely use journal articles, research reports and hospital libraries as the basis for their practice [9,11]. Similar trends have been noticed among community nurses [12]. A systematic review of 20 articles emphasized that while community nurses displayed positive attitudes towards EBP, this did not necessarily translate into practice and their level of knowledge about EBP was found to be insufficient [10].

The reduced implementation of EBP in daily nursing practice is related to impediments to which the specific procedure is subject. There is a lot of evidence concerning the barriers and facilitators for EBP implementation [13,14,15]. Concerning nurses working in hospital settings, a literature review concludes that there are both organizational and individual barriers to implementing EBP [16]. There is also evidence regarding the implementation of EBP by community nurses in the community and primary care [6,10,17,18]. In this sector, several facilitators and barriers were identified, emphasizing the intricate relationship between service context and individual factors in achieving successful implementation, along with managerial support. In a systematic review, the authors [6] concluded that the adoption of an innovation by community nurses depends on their decision-making process. This decision is positively influenced by several factors, including the practicality of the innovation in terms of time and cost-effectiveness, ease of use, adaptability in an autonomous and flexible manner and its impact on patient–nurse relationships and trust, care quality, working conditions and professional development. Community nurses identified organizational infrastructure and change as significant barriers. A previous study concluded that hospital and community nurses encounter similar barriers and facilitators and that their levels of EBP skills and knowledge were comparable [19]. Education is recognized as both a barrier and a facilitator for the implementation of EBP by nurses. In almost all studies, the primary strategy for overcoming these barriers is further training and education of nurses [20]. 

To our best knowledge, Greek nurses’ knowledge, attitudes and implementation of EBP [21,22], especially in community nurses [23], is understudied. In particular, it was found that the majority of the participants did not have an in-depth knowledge of EBP and did not implement it in clinical practice [21]. In another study [24], in a sample of midwives and midwifery students, 43.5% of the participants stated that they knew the term EBP, with the first source of information being colleagues (52.2%) and the second searching the internet in general (48.8%), but not in EBP databases. Schesaki et al. [22], in a recent study, indicated that factors such as holding a master’s degree, having experience in writing academic papers, working in a university clinic and possessing computer skills had a positive influence on hospital nurses’ attitudes, knowledge, skills and practices. In the only study involving a sample of community nurses, it was revealed that approximately half of these nurses were acquainted with EBP and held favorable attitudes toward it. However, the study also highlighted a noteworthy degree of uncertainty regarding the effective implementation of EBP [23]. 

Although every country, including Greece, faces similar challenges when it comes to implementing EBP among nursing professionals, studying this issue in Greece is particularly important due to several significant changes in nursing and community nursing in recent years. Since 2018, all technological nursing schools in Greece have become universities. Consequently, new curriculums have been introduced in nursing schools, and numerous postgraduate programs have been offered to nursing graduates. This includes the establishment of master’s programs in community nursing. Moreover, the “Public health/Community nursing” speciality was introduced. Additionally, the present study was conducted during the pandemic period, which created new arrangements and tasks for community nurses [25]. Knowing the “enemy” (COVID-19) was translated to knowing the evidence and implementing it, which was the first step towards its prevention and management. A large number of registered nurses staffed the understaffed Greek primary healthcare services. Approximately 4000 registered nurses were hired to staff mainly primary healthcare and intensive care units around the country during the pandemic [26]. All of these changes are expected to contribute to improving the knowledge of nurses, especially community nurses, regarding EBP. Thus, the researchers of this study have found it significantly important to study this specific issue in Greece, considering all the changes that have been implemented in a relatively short period of time. So, it would be possible to evaluate whether this widely available academic knowledge contributes to an equally profound implementation of EBP in everyday clinical practice and to compare the results of this study with previous ones conducted nationally and internationally.

Additionally, EBP is a global concern within the nursing scientific community. However, the existing literature primarily focuses on nurses in hospital settings. Community nurses, who constitute a substantial portion of the nursing workforce in a distinct dynamic and complex work environment, have received comparatively less attention. Therefore, for the above reasons, investigating community nurses’ attitudes, knowledge and EBP practices and factors that affect them is a valuable endeavor representing the unique contribution of our study.

In this study, we considered community nurses to mean the nurses who provide healthcare in the community such as home healthcare, school and occupational health, and nurses working in health centers and other healthcare community services. The aim of this study was to investigate factors that influence the attitudes, knowledge/skills and practices of nurses working in primary healthcare in relation to EBP.

## 2. Materials and Methods

### 2.1. Study Design and Sample

A cross-sectional study was conducted with a convenience sample in which 164 community nurses participated from all over Greece. The questionnaires were completed by the participants on a free online platform during the period February 2022–April 2022. The two researchers (TA & EN) emailed the questionnaire link to their personal networks of community nurses. They were asked to share the link with colleagues working in community services in health centers or other settings, such as schools, home healthcare, occupational health, etc. The data were collected in an Excel form, downloaded by the researchers and subsequently analyzed. During this period, there were strict restrictions on access to healthcare services and dissemination of printed materials due to the COVID-19 pandemic.

The inclusion criteria were as follows: (a) being a registered nurse, and (b) working in community services. Nursing assistants working in the community were excluded from the study because their education does not include an EBP approach. It is important to note that in Greece, there are two levels of nursing education. University-level nursing education leads to a registered nurse’s bachelor’s degree, while a lower level of education leads to nursing assistants. EBP is a subject taught in higher education, following international scientific nursing curriculums, and is relevant to the responsibilities, roles and competencies of registered nurses.

The minimum sample size required 156 nurses considering the low effect size (f^2^ = 0.08), the precision level of 5% (alpha level), the power of 95% and the number of independent variables of eight [27]. Our sample included 164 nurses. 

### 2.2. Measures and Data Collection

An anonymous, self-completed questionnaire, which consisted of two parts, was used to collect the data. 

The first part of the questionnaire consisted of the Evidence-Based Practice Questionnaire created by Upton and Upton [28]. This comprises of 24 questions that measure nurses’ practice, attitude and knowledge/skills regarding EBP. Items are scored on a 7-point scale (1–7) and divided into 3 distinct subscales: 6 questions about EBP practice, 4 questions about attitudes toward EBP and 14 questions about EBP knowledge/skills. We calculated the total score for each dimension by adding the answers in the dimension questions and dividing by the total number of answers. For instance, for the EBP practice dimension, we added the answers on the 6 questions and we divided this total sum by six. Thus, scores on each dimension took values from 1 to 7. Higher values indicated a higher level of EBP practice and knowledge/skills and more positive attitudes toward EBP. The Greek version of the tool had an internal consistency of 0.95 for the subscale of “practice”, 0.72 for the subscale of “attitudes” and 0.92 for the subscale “knowledge and skills” about EBP [29]. 

The second part consisted of the demographic and working characteristics of the nurses (gender, age, education level, time worked in the community, total employment time, workplace, work position, participation in an educational seminar in the last year and presentation of a paper at a conference in the last year).

### 2.3. Ethical Considerations

Permission was granted from the Ethics Committee of the institution (protocol No: 17363-23/02/2022) in order to carry out the study. Participants were informed about the aim of the study and about maintaining anonymity, as well as the possibility of exiting the study at any point if they so wished.

### 2.4. Statistical Analysis

We use numbers and percentages to present categorical variables. Additionally, we use mean, standard deviation, median, minimum value and maximum value to present continuous variables. We evaluated distribution of continuous variables using the Kolmogorov–Smirnov test and Q-Q plots. We found that the “practice” variable did not follow normal distribution, while the other continuous variables followed normal distribution. First, we examined the relationship between socio-demographic variables and the attitudes, knowledge and practice of community nurses regarding EBP using bivariate analysis. In particular, we used an independent samples *t*-test (to compare a continuous variable on two independent groups), analysis of variance (ANOVA) (to compare a continuous variable in ≥2 independent groups), Pearson’s correlation coefficient (to explore the correlation between two continuous variables that followed the normal distribution) and Spearman’s correlation coefficient (to explore the correlation between two continuous variables that did not follow the normal distribution). We did not include the working position in bivariate analysis since there were only ten head nurses and the results would otherwise be unstable. Then, we applied multivariable linear regression models to eliminate confounding factors. In particular, independent variables that were significantly different (*p* < 0.20) in bivariate analyses were entered into backward stepwise multivariable linear regression models. In that case, we chose to use the cut-off point of 0.20 instead of 0.05 to reduce the likelihood of not including statistically significant variables later in the multivariable analysis [30,31]. We used the scores on attitudes, knowledge and practice of community nurses regarding EBP as the dependent variables. In these cases, we present adjusted coefficient beta, 95% confidence intervals (CI) and *p*-values. *p*-values less than 0.05 were considered statistically significant. We used the IBM SPSS 21.0 for the analysis.

## 3. Results

In the present study, 164 nurses participated, the majority being women (81.1%). The mean age of the participants was 41.6 ± 9.3 years and 42.7% had a postgraduate degree. The mean time of community service was 9.1 ± 7.9 years, almost half (55.5%) had attended an educational training seminar in the previous year and 18.3% had presented a paper at a conference in the last year (Table 1).

Descriptive characteristics of the EBPQ subscales showed a high level of “attitudes” (mean 5.5 ± 0.9) and “knowledge/skills” (mean 5.5 ± 0.7) in the corresponding subscale of the EBPQ and an average level of EBP practice in the “practice” subscale (mean 4.5 ± 1.5) (Table 2). All correlations between the three subscales were statistically significant: (a) knowledge/skills and practice (r = 0.29, *p* < 0.001), (b) knowledge/skills and attitudes (r = 0.59, *p* < 0.001) and (c) practice and attitudes (r = 0.16, *p* = 0.04). Correlations between knowledge/skills and practice, and practice and attitudes, were weak, while between knowledge/skills and attitudes the correlation was moderate.

In the bivariate relationships between the independent variables and the total score in each subscale, it was found that the subscales “knowledge/skills” and “practice” of the EBPQ were statistically significantly related with the higher level of education of the participants (*p* = 0.001 and *p* = 0.002, respectively), with participation in an educational training seminar (*p* = 0.003 and *p* = 0.05, respectively) and with participation in a conference with a presentation in the last year (*p* = 0.01 and *p* = 0.02, respectively) (Table 3).

Finally, the results of the multivariable linear regression analysis showed that health professionals with a higher educational level implemented EBP in their practice more often (*p* = 0.005) compared to those with a lower level of education. Furthermore, they demonstrated a greater level of “knowledge and skills” about EBP (*p* = 0.003). Additionally, an increase in the knowledge/skills score was associated with a more positive “attitude” towards EBP (*p* < 0.001) and better practice of EBP (*p* = 0.003) (Table 4).

All models were adjusted for gender, age, workplace in the community, participation in an educational seminar in the last year, participation in a conference with a paper presentation in the last year, working experience in the community setting and total years of working experience. 

## 4. Discussion

The present study emphasized the vital role of education in enhancing the EBP competence of community nurses. Specifically, the participants’ level of education was identified as the primary factor related to the “knowledge/skills” and “practice” of EBP in Greek community nurses. Additionally, the “knowledge/skills” score was found to impact both the “attitudes” and “practice” scores of EBP.

In particular, a positive correlation was found between knowledge/skills and attitudes and practice regarding EBP. Specifically, an increase in knowledge/skills regarding EBP of community nurses could also improve their attitudes and their practice regarding EBP. This interplay between knowledge/skills, attitude and practice is pivotal for increasing the utilization of EBP among healthcare professionals. It underscores the importance of enhancing healthcare professionals’ understanding and competence in EBP in order to foster positive attitudes and propel the practical application of EBP in clinical settings. These findings highlight the significance of educational interventions and training programs to strengthen knowledge/skills, promote positive attitudes and facilitate the successful implementation of EBP. A qualitative study also concluded that primary healthcare nurses demonstrated a favorable attitude toward EBP. However, its infrequent utilization was attributed to insufficient competence and a lack of organizational support for its implementation [32]. Gerrish et al. paid attention to community nursing skills development in accessing and reviewing research information [33].

The implementation of educational programs in order to improve community nurses’ knowledge and consequently to increase their attitudes and use of EBP constitutes a challenge. A randomized controlled pilot trial revealed that the intervention group of health visitors, after an EBP educational intervention program, showed stronger beliefs and higher implementation of EBP as well as increased group cohesion and reduced attrition or turnover versus the control group [34]. A recent meta-analysis of six randomized controlled trials and four clinical controlled trials concludes that educational interventions have demonstrated their effectiveness in enhancing nurses’ EBP knowledge, skills, attitudes, confidence and behaviors. It proposes the integration of EBP education interventions to be an integral component of nurses’ ongoing professional development within clinical settings and summarizes a combination of ways that these interventions should take place (lectures, group discussions and hands-on practice in face-to-face and online formats) [35]. Additionally, the need for long-term support of the participants in further educational interventions was noted due to the fact that long-term effectiveness is still under study [6,10]. In a community clinical setting, the effectiveness of training on the implementation of evidence was also confirmed. For example, it was found that diabetes mellitus (DM) educational programs improved nurses’ practices in glycemic self-monitoring promotion, patient education on DM self-care, patient follow-up and referral, medication management, goal setting and addressing family and social issues [36]. Knowledge acquisition was proposed as a main strategy for overcoming knowledge deficits of nurses concerning DM management [37].

Another finding of this study was that health professionals with postgraduate studies (42.7%) had more knowledge about EBP and implemented it in their practice more often than those who had only a bachelor’s degree. The present finding is in agreement with previous studies. A Finnish study states that community nurses with a master’s degree (16.8%) knew and implemented EBP very well [38]. Braid and Miller’s study found that community nurses with master’s degrees had more skills regarding EBP, assessing and reviewing articles and synthesizing literature information. Interestingly, their study revealed differences between community nurses’ engagement with EBP and nurses in a clinical setting. More specifically, public health nurses were found to be more engaged with EBP compared to home healthcare and primary healthcare nurses. Researchers attributed this finding to the organizational culture of each clinical environment [39]. Our study did not reveal that kind of difference. Maybe a larger sample of community nurses from each setting would disclose new relationships between these nurses’ characteristics. There is also a strong body of evidence supporting the positive relationship between a high level of education of nurses in acute hospital settings and the knowledge and implementation of EBP [22,40,41,42]. In a recent Greek study, the postgraduate qualification of hospital nurses was associated with a high level of competence related to EBP [22], and a study in Malawi [42] revealed a positive relationship between a postgraduate level of study (14.2%) with the knowledge and implementation of EBP. Conversely, the lack of correlation between the practice of EBP and the high educational level of nurses and midwives working in hospitals in Ethiopia was attributed to the fact that there was no clear division of duties between higher and lower educational levels [9]. A study of Greek midwives showed that a high educational level positively influenced attitudes towards EBP [24], a result reinforced by other studies [43,44]. A recent study concluded that master’s graduates (14%) were more skilled with regard to the practice of EBP [45]. 

In some countries, where EBP has been part of the curriculum for years, more experienced nurses show higher EBP knowledge, attitudes and implementation [46]. Similar findings in other European studies link greater EBP knowledge and implementation to including EBP courses in the curriculum [44,47]. Low- and middle-income countries tend to under-implement EBP [48]. Nursing schools should foster a culture of EBP in both theoretical and clinical education [49,50], as seen in Greek universities. Future research could be interesting to investigate the cultural impact on EBP knowledge, attitudes and implementation across healthcare levels.

Another finding of our study was the high percentage of participants (37.8% and 4.9%) who were master’s and doctoral graduates. According to a national study, the respective percentages represented 14% and 0.82% of all nurses in the National Health System (15,833 nurses) in Greece, while nurses working in health centers seem to constitute only 2.17% of all nurses [51]. These findings may be attributed to the greater awareness of postgraduate nurses participating in the study due to convenience sampling compared to non-postgraduate nurses. Postgraduate studies, which include advanced research methodology courses [52], require knowledge and application of recent evidence-based data for completion [42]. They also promote participation in conferences for the dissemination of new data. Furthermore, postgraduate studies and lifelong learning contribute to expanding knowledge, often challenging previous knowledge and practices. Therefore, improving the implementation of EBP among nurses can be achieved through training, either individually or as part of a curriculum [53].

The present study showed that participants who attended seminars or had a conference presentation during the last year had more knowledge/skills about EBP and implemented it more often than those who did not in the last year. This aligns with the findings of Schesaki et al. [22], who noted that Greek hospital nurses with academic writing experience in the previous five years exhibited higher attitudes, knowledge and skills. A previous study mentioned that difficulty in attending seminars led to challenges in implementing EBP [54]. Similarly, another study found that community nurses’ familiarity with EBP did not translate into interest in implementation, often attributed to a lack of seminar engagement [23]. Generally speaking, conferences and seminars serve as platforms for new scientific data, enriching participants’ knowledge. This aids healthcare professionals in becoming research-oriented, blending research and experience. Creating conference presentations demands rigorous preparation, involving sourcing new knowledge and citing value-adding references. Healthcare professionals’ participation in these events showcases their expertise in EBP and practical commitment to staying current in their field.

In this study, healthcare professionals had a positive attitude and knowledge/skills about EBP, as indicated by mean scores, but this did not always translate into practice. Similar findings of poor implementation of EBP emerged in other studies on community nurses [10,46,55]. The lowest mean score related to the “practice” subscale, particularly the question “Did you critically appraise any article you found in the literature based on defined criteria?”, while the highest mean score was in the “attitudes” subscale, specifically the statement that “Evidence-based practice is fundamental to professional practice”. This suggests that Greek community nurses may face challenges in implementing EBP in practice, despite their high levels of knowledge/skills and positive attitudes. Overall, nurses encounter obstacles related to organization, EBP communication and research [16,56,57]. A systematic review [10] cites time and resource constraints as primary barriers to translating community nurses’ knowledge and attitudes into EBP practice. 

This study was conducted during the public health crisis created by COVID-19. A recent comparative study regarding pre- and post-lockdown competences and barriers towards EBP by healthcare professionals revealed a significant increase in “beliefs and attitudes” and “evaluation” of EBP in the post-lockdown group [7]. During the pandemic, healthcare professionals were continuously updating their knowledge while also assessing the quality and reliability of the available information. This information affected their attitudes toward the virus. A study on Greek primary care physicians found that greater levels of positive attitudes towards COVID-19 were associated with increased likelihood of engaging in higher levels of practices towards its prevention [58]. Generally speaking, the arrival of COVID-19 and the measures required to contain its transmission underscore the significance of comprehending and implementing evidence-based decision making in the practice of every healthcare professional.

Our study is subject to limitations. It was a cross-sectional study, which did not allow for the exploration of cause–effect relationships between variables. Thus, more valid studies such as cohort studies could add invaluable information on the factors that influence the attitudes, knowledge/skills and practices of nurses. The study used a convenience sample due to the restrictive measures imposed during the COVID-19 pandemic in the country and primary healthcare services. The online completion of the questionnaire also possesses selection biases concerning the validity of the participants’ characteristics. In our study there was an over-representation of female gender. This gender imbalance is a reality in the nursing profession in Greece. Additionally, quantitative research does not allow participants to deviate from the questions and express or justify their perceptions, ideas or practices. We should carefully consider the generalization of the study results due to the above limitations, as well as the fact that this study was conducted during the pandemic where issues concerning EBP and its implementation concerning COVID-19 were questioned by the national and international scientific community. Thus, a random and larger sample under normal everyday work in the primary healthcare services and not under the pressure that the COVID-19 pandemic had posed might have allowed a more secure generalization of our results. Nevertheless, despite these limitations, the present study provides an understanding of knowledge, practice and attitudes towards EBP among Greek registered nurses. 

## 5. Conclusions

The study findings emphasize the significance of giving priority to the proficiency of healthcare professionals in EBP, through education and training, which has emerged as the pivotal factor related with their practice, attitudes and EBP knowledge/skills. Initiating EBP education at the undergraduate level to raise awareness among students is a foundational step. Subsequently, promoting skill development in EBP at the professional level through incentives is essential. Moreover, organizations should establish practice environments equipped with the necessary infrastructure for EBP adoption and implementation. Given the evolving nature of healthcare sciences, continuous high-level education in EBP and lifelong learning for healthcare professionals are imperative to enhance service quality. 

## Figures and Tables

**Table 1:** Demographic and employment characteristics of the participants.

Characteristics	Ν = 164	%
Gender		
Male	31	18.9
Female	133	81.1
Age (mean ± SD)	41.6 ± 9.3	
Educational level		
Higher education	94	57.3
Master’s degree	62	37.8
PhD	8	4.9
Workplace in the community-based setting		
Local primary healthcare setting (ΤOΜΥ)	20	12.2
Health center	97	59.1
Outpatient clinic	7	4.3
Home healthcare	7	4.3
School	8	4.9
Mental health unit	8	4.9
Occupational health nurse	4	2.4
Other local primary healthcare setting	9	5.5
Working position		
Clinical staff nurse	154	93.9
Head nurse	10	6.1
Years of working experience in the community setting (mean ± SD)	9.1 ± 7.9	
Total years of working experience (mean ± SD)	13.8 ± 9.2	
Participation in a conference with a paper presentation in the last year		
Yes	30	18.3
No	134	81.7
Participation in an educational seminar in the last year		
Yes	91	55.5
No	73	44.5

SD: Standard deviation.

**Table 2:** Descriptive statistics of the three EBPQ subscales “practice”, “attitudes” and “knowledge/skills” (N = 164).

EBPQ Subscale	Mean	SD	Median	Min Value	Max Value
Practice of evidence-based practice	4.5	1.5	4.8	1	7
Attitudes towards evidence-based practice	5.5	0.9	5.5	2	7
Knowledge/skills associated with evidence-based practice	5.5	0.7	6	3.6	6.9

EBPQ: Evidence-Based Practice Questionnaire; SD: standard deviation; Min: minimum; Max: maximum.

**Table 3:** Bivariate analysis between independent variables and the total score on the EBPQ subscales of “knowledge/skills”, “attitudes” and “practice” (N = 164).

	Practice of EBP			Attitudes towards of EBP			Knowledge/Skills Associated with EBP		
Independent Variable	Mean	SD	*p*	Mean	SD	*p*	Mean	SD	*p*
Gender			0.2 ^a^			0.4 ^a^			0.4 ^a^
Male	4.8	1.5		5.7	0.8		5.6	0.8	
Female	4.4	1.5		5.5	0.9		5.5	0.7	
Age		−0.03 ^b^	0.7 ^b^		0.1 ^b^	0.1 ^b^		0.03 ^b^	0.9 ^b^
Educational level		0.3 ^c^	0.001 ^c^		0.1 ^c^	0.4 ^c^		0.3 ^c^	0.002 ^c^
Workplace in the community			0.2 ^d^			0.4 ^d^			0.2 ^d^
Local primary healthcare setting (ΤOΜΥ)	4.9	1.3		5.4	0.8		5.3	0.7	
Health Centers	4.3	1.5		5.5	0.9		5.4	0.7	
Other	4.7	1.5		5.7	0.9		5.6	0.6	
Participation in an educational seminar in the last year			0.003 ^a^			0.9 ^a^			0.05 ^a^
Yes	4.8	1.4		5.5	0.9		5.6	0.7	
No	4.1	1.6		5.6	0.8		5.4	0.7	
Participation in a conference with a paper presentation in the last year			0.01 ^a^			0.9 ^a^			0.02 ^a^
Yes	5.1	1		5.5	0.7		5.7	0.7	
No	4.4	1.5		5.5	0.9		5.4	0.7	
Working experience in the community setting		0.1 ^c^	0.1 ^c^		0.1 ^c^	0.1 ^c^		0.01 ^c^	0.8 ^c^
Total years of working experience		0.1 ^c^	0.6 ^c^		0.1 ^c^	0.3 ^c^		0.01 ^c^	0.6 ^c^

^a^ independent samples *t*-test; ^b^ Pearson correlation coefficient; ^c^ Spearman correlation coefficient; ^d^ analysis of variance; EBPQ: Evidence-Based Practice Questionnaire; *p*: *p*-value; TOMY: local primary healthcare setting.

**Table 4:** Multivariable linear regression analysis with the dependent variables of the total score on the EBPQ subscales of “knowledge/skills”, “attitudes” and “practice” (N = 164).

Independent Variable	Unstandardized b Coefficient	95% Confidence Interval for Coefficient b	Standardized b Coefficient	*p* Value	R^2^ (%)
Dependent variable: Subscale “Practice of EBP”					11.7
Educational level	0.5	0.2 to 0.9	0.1	0.005	
Knowledge/skills score	0.5	0.2 to 0.8	0.6	0.003	
Dependent variable: Subscale “Attitudes towards of EBP”					35.5
Knowledge/skills score	0.8	0.6 to 0.9	0.6	<0.001	
Dependent variable: Subscale “knowledge/skills associated with EBP”					6.3
Educational level	0.3	0.1 to 0.5	0.3	0.003	

## Data Availability

Data are contained within the article.
